# Effects of grass species and grass growth on atmospheric nitrogen deposition to a bog ecosystem surrounded by intensive agricultural land use

**DOI:** 10.1002/ece3.1534

**Published:** 2015-06-03

**Authors:** Miriam Hurkuck, Christian Brümmer, Karsten Mohr, Oliver Spott, Reinhard Well, Heinz Flessa, Werner L Kutsch

**Affiliations:** 1 Thünen Institute of Climate-Smart Agriculture, Federal Research Institute for Rural Areas, Forestry and FisheriesBundesallee 50, Braunschweig, 38116, Germany; 2 Landwirtschaftskammer NiedersachsenMars-la-Tour Str. 1-13, Oldenburg, 26121, Germany; 3 Department of Soil Physics, Helmholtz Centre for Environmental Research - UFZTheodor-Lieser-Straße 4, Halle, 06120, Germany; 4 Integrated Carbon Observation System (ICOS), University of HelsinkiHeadoffice, FI-00014, Finland

**Keywords:** ^15^N isotope dilution technique, biomonitoring, critical load, *Eriophorum vaginatum*, integrated total nitrogen input, *Lolium multiflorum*, nitrogen deposition, ombrotrophic bog

## Abstract

We applied a ^15^N dilution technique called “*Integrated Total Nitrogen Input”* (ITNI) to quantify annual atmospheric N input into a peatland surrounded by intensive agricultural practices over a 2-year period. Grass species and grass growth effects on atmospheric N deposition were investigated using *Lolium multiflorum* and *Eriophorum vaginatum* and different levels of added N resulting in increased biomass production. Plant biomass production was positively correlated with atmospheric N uptake (up to 102.7 mg N pot^−1^) when using *Lolium multiflorum*. In contrast, atmospheric N deposition to *Eriophorum vaginatum* did not show a clear dependency to produced biomass and ranged from 81.9 to 138.2 mg N pot^−1^. Both species revealed a relationship between atmospheric N input and total biomass N contents. Airborne N deposition varied from about 24 to 55 kg N ha^−1^ yr^−1^. Partitioning of airborne N within the monitor system differed such that most of the deposited N was found in roots of *Eriophorum vaginatum* while the highest share was allocated in aboveground biomass of *Lolium multiflorum*. Compared to other approaches determining atmospheric N deposition, ITNI showed highest airborne N input and an up to fivefold exceedance of the ecosystem-specific critical load of 5–10 kg N ha^−1^ yr^−1^.

## Introduction

One of the main threats to the diversity, functioning, and species composition of most natural and semi-natural aquatic and terrestrial ecosystems is increasing nitrogen (N) input via atmospheric deposition (Rockström et al. 2009).

Atmospheric N is deposited as sedimenting and nonsedimenting particles and gases in both dry and wet processes on soil and plant surfaces (see VDI[Bibr b46] 4320, Part 1). Depending on different properties of the site (e.g., meteorological conditions, vegetation, N deposition), the share of dry deposition on plant surfaces changes. Gaseous NO_x_ and NH_3_, as well as 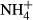 and 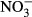, are taken up from the atmosphere through stomata and cuticles. While N uptake from soils is metabolically driven and plants take up N according to demand, foliar N uptake cannot be controlled and gases exchange as soon as stomata open (Schulze et al. 2005). However, depending on the stomatal compensation point (function of 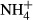 concentration and pH value of plant apoplasts), NH_3_ can be either taken up or emitted by plants (Mattsson and Schjørring 2003). Both soilborne 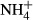 and 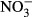 are assimilated into amino acids and are transported to the plant leaves and shoots. Airborne NH_3_, however, can be either assimilated or accumulated as amino acids dependent on the ratio of NH_3_ uptake and NH_3_ assimilation rate (Guderian 2001; Schulze et al. 2005). Ammonia accumulation within the plant cells can result in growth reduction, an increased sensitivity to, for example, frost and may even cause toxic effects such as alkali burning of plant tissue and subsequent necrosis (Van der Eerden 1982; Pearson and Stewart 1993; Krupa 2003).

High levels of N deposition can have pronounced effects on primary production, especially in nutrient-poor ecosystems. Ombrotrophic bogs are N-limited ecosystems and belong thus to the most sensitive ecosystems to increased atmospheric N input (Succow and Joosten 2001). Nitrogen input into bogs occurs by precipitation, dry deposition, and biological N_2_ fixation. Critical N loads have been estimated to range from 5–10 kg N ha^−1^ yr^−1^ (Bobbink and Roelofs 1995; Tomassen et al. 2003; Bobbink et al. 2010). As has been shown in various studies in different bog ecosystems across Europe, exceedance of this threshold is expected to result in a shift in plant species compositions with decreases in plant diversity, a reduction in plant species numbers and invasion by more N-demanding species (i.e., *Molinia caerulea*, *Betula pubescens*) (Mountford et al. 1993; Bobbink et al. 1998; Clark and Tilman 2008; Verhoeven et al. 2011).

Conventional methods to monitor wet or bulk N deposition (i.e., by wet only and bulk samplers) as well as gaseous N deposition (i.e., by passive samplers, denuder-filter systems) consider only parts of the total atmospheric N input. Furthermore, these methods do not take into account direct plant uptake of atmospheric N. Biomonitoring approaches overcome this shortcoming by using ^15^N-labeled monitors (soil–plant systems, e.g., Böhlmann et al. 2005; Böhme et al. 2002; He et al. 2007; Russow and Weigel 2000; Sommer 1988). The ^15^N tracer is circulated in those pot systems and only dilutable by atmospheric N input, thus providing a direct measurement of total atmospheric N input into soil, plant, and solution covering dry and wet deposition as well as direct N uptake by plants over the exposure period. In 1995, Mehlert et al. developed the *Integrated Total Nitrogen Input* (ITNI) approach. This ^15^N biomonitor technique has been applied in several field experiments in Germany and China using varying monitor plants (Russow and Weigel 2000; Weigel et al. 2000; Böhme et al. 2002, 2003; Böhlmann et al. 2005; Russow and Böhme 2005; He et al. 2007, 2010b). The results show that deposition and plant uptake of atmospheric N increase with increasing biomass production and leaf area of the monitor plant (e.g., Böhme et al. 2003; Russow and Böhme 2005). While ITNI is the only known technique that measures total atmospheric N deposition including wet inorganic and organic N, gaseous N, and direct N uptake by plants, the methodological uncertainties of this biomonitoring approach yet need to be discussed.

In this study, we investigated total airborne N input into biomonitors exposed at a semi-natural raised bog in northwestern Germany. We used the ITNI approach with *Lolium multiflorum* and *Eriophorum vaginatum* differing in N demands as monitor plants. Furthermore, various levels of fertilizer were applied to change biomass and N status of the monitor plants. We hypothesized that (1) local atmospheric N depositions from intensive agricultural land use exceed the ecosystem-specific critical load of 5–10 kg N ha^−1^ yr^−1^, (2) plant biomass production positively correlates with airborne N uptake, (3) increasing plant N status from increased N addition leads to saturation of N within plant cells and thus decreasing atmospheric N uptake by the monitor plant, and (4) ITNI results in higher airborne N input into the study site than conventional approaches as it considers direct N uptake by plants.

## Materials and Methods

### Site description

The experimental work was conducted in the ombrotrophic bog “Bourtanger Moor” in northwestern Germany. The study site (52°39′21.25″ N, 7°11′0.17″ E, 19 m a.s.l.) is located within a protected bog area (size 5 ha). It is surrounded by extensively drained and excavated peat areas. The region is further characterized by intensive crop production and livestock breeding.

After ground water lowering, peat excavation, and renaturation, the recent vegetation comprises bog heather (*Erica tetralix*), purple moor-grass (*Molinia caerulea*), cotton grass (*Eriophorum vaginatum*, *Eriophorum angustifolium*), coppices with birches and Scots pines (*Betula pubescens, Pinus sylvestris*), and *Sphagnum* mosses.

The annual average air temperature is about 9.5°C, and annual precipitation is about 751 mm (1981–2010; German Meteorological Service, Emden, Germany, distance to study site ca. 80 km). During the experimental period from 2011 to 2013, annual average air temperature was 10.5°C and annual precipitation was 781 mm (own meteorological observations). Mean annual water level was around 0.1 m below surface with considerable seasonal variation (constantly saturated soil in December and January and 0.4 m below surface in September). The maximum peat layer depth is approximately 4 m.

A detailed description of the historical development of the peat bog and livestock characteristics can be found in Casparie (1993) and Hurkuck et al. (2014).

### Experimental design

#### Experimental setup

To determine the total airborne N input into the investigated area, an *Integrated Total Nitrogen Input* (ITNI) approach was used (Russow and Weigel 2000; Weigel et al. 2000; Russow and Böhme 2005; Tauchnitz et al. 2010). This method allows the quantification of direct atmospheric N input to a soil–plant model system based on a ^15^N pool dilution technique. The biomonitor is supplied with ^15^N-labeled tracer solution and airborne N input into the ITNI system – consisting of 99.6% of ^14^N – dilutes the ^15^N label according to the atmospheric N deposition rate. In 2011, *Lolium multiflorum* Lam. (var. Lema) was used as ^15^N-labeled biomonitor plant. In 2012, two different grass species, the fast-growing crop grass *Lolium multiflorum* Lam. (Lema) and the well-germinating site-specific species *Eriophorum vaginatum*, were exposed as monitor plants at our experimental site.

**Figure 1:**
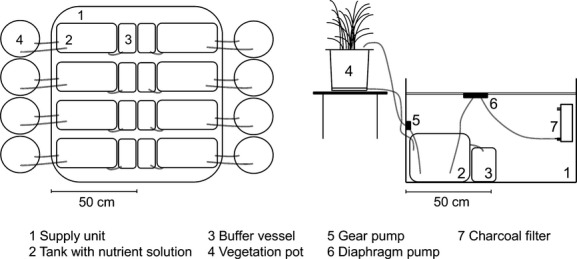
Scheme of the integrated total nitrogen input (ITNI) setup in the field: overview (left) and cross section (right).

Germination of grass seeds was carried out on N-free quartz sand in a glasshouse. Plants were supplied with a ^15^N tracer solution. After about four and six weeks of precultivation, eight *Lolium multiflorum* or sixteen *Eriophorum vaginatum* seedlings were planted on about 10-kg N-free quartz sand in Kick-Brauckmann plant culture pots (surface area 0.038 m²) (STOMA GmbH, Siegburg, Germany), respectively (cf. Tables S1, S2, S3). In total, five and eight pots per species were prepared and exposed in the study area in July 2011 and May 2012, respectively (Fig.1). About 600 mL nutrient solution from a tank (total volume of solution 15 L) was given automatically to the vegetation pot by a gear pump twice a day. The nutrient solution contained macronutrients (34.021 mg L^−1^ KH_2_PO_4_, 130.693 mg L^−1^ K_2_SO_4_, 160.205 mg L^−1^ MgSO_4_·7 H_2_O, 13.514 mg L^−1^ FeCl_3_·6 H_2_O) and micronutrients (0.061 mg L^−1^ H_3_BO_3_, 0.025 mg L^−1^ CuSO_4_·5 H_2_O, 0.169 mg L^−1^ MnSO_4_·H_2_O, 0.001 mg L^−1^ Na_2_MoO_4_·2 H_2_O, 0.288 mg L^−1^ ZnSO_4_·7 H_2_O) following He et al. (2007). ^15^N-labeled N fertilizer was applied manually two to six times over the growing season in the form of dissolved ^15^NH_4_^15^NO_3_ (5.431 at. % ^15^N (2011), 5.521 at. % ^15^N (2012), conc. 20 g N L^−1^) (Campro Scientific GmbH, Berlin, Germany). Excessive nutrient solution was returned to the tank and one buffer vessel per vegetation pot was set up to collect possible overflow water during heavy rain events. The solution was ventilated with air sucked through charcoal filter by a diaphragm pump to prevent gaseous N losses from the nutrient solution by anoxic microbial processes (i.e., denitrification) (Fig.1).

#### Nitrogen fertilization

In 2011, each of the five pots with *Lolium multiflorum* was supplied with 25 mL ^15^N tracer solution (=0.50 g N pot^−1^). The 2011 data were used to quantify total airborne N input into the biomonitor systems. To study the effects of grass species and grass growth on total atmospheric N deposition, we applied different levels of fertilizer by varying doses of ^15^N tracer solution to change biomass and N status of the monitor plants during the experiments in 2012. For this purpose, three pots with *Eriophorum vaginatum* received a low and a high N supply of 0.10 g N pot^−1^ and 0.44 g N pot^−1^, respectively. Two pots were moderately (0.26 g N pot^−1^) fertilized. According to the experiments in 2011, three pots with *Lolium multiflorum* were fertilized with 0.50 g N pot^−1^. Additionally, two pots with moderate (0.40 g N pot^−1^) and three pots with low N supply (0.30 g N pot^−1^) were exposed in the field. After exposure times ranging from 86 to 93 days (2011) and 147 to 156 days (2012) at our experimental site (cf. Tables S1, S2 and S3), the vegetation pots were collected for harvest at the end of October in 2011 and in 2012. One high and one low fertilized pot per species remained in the field until end of May 2013 to provide data for a whole year of investigation. To our knowledge, this is the first attempt to quantify annual atmospheric N deposition using the ITNI approach. Previous studies extrapolated results obtained from ITNI experiments during the growing season to a whole year (cf. Section Analyzes; e.g., Böhlmann et al. 2005; Russow and Böhme 2005).

#### Analyzes

For analysis, the different parts of the biomonitor system were separated into aboveground biomass, roots, substrate (quartz sand), and nutrient solution. Separation of roots from substrate was carried out by sieving the sand with 3 L slightly acidic (sulfuric acid, H_2_SO_4_) potassium chloride (KCl) solution (0.02 mol/L) (sieve mesh size 500 *μ*m). Total N content and ^15^N abundances of plant material were analyzed using an isotope ratio mass spectrometer (Delta C, Finnigan GmbH, Bremen, Germany) coupled to an elemental analyzer (NC 1108, Carlo Erba Instruments Ltd., Wigan, UK). The measurement uncertainty of total N analysis was calculated as the deviation between measured and theoretical N contents of control measurements (*n* = 12) and was 2.1%. Determination of N contents of substrate was conducted using the Kjeldahl method (10 g substrate, *n* = 3) with subsequent water steam distillation (Faust et al. 1981). ^15^Nitrogen abundances of total N in distilled substrate samples (i.e., 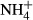) were measured by the SPINMAS technique (Sample Preparation unit for Inorganic Nitrogen (SPIN) coupled to a MAss Spectrometer (MAS); Stange et al. (2007)). Total N contents and ^15^N abundances of solution were analyzed by oxidizing solute N completely to 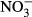 prior to the SPINMAS analyzes. For that purpose, 3 mL of solution was treated with 1 mL potassium persulfate (K_2_S_2_O_8_) solution (0.15 mol/L) and 50 *μ*L sodium hydroxide (NaOH) solution (2.5 mol/L) and incubated in a compartment drier for 48 h at 80°C. Measurement uncertainties related to SPINMAS analysis were in the order of <1% (^15^N analysis) and <5% (total N analysis).

### Calculation of airborne N deposition and ^15^N recovery

Calculation of total N deposition into the vegetation pots was performed according to Russow and Weigel (2000):

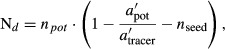
1where N_d_ is the total atmospheric N deposition into the soil–plant model system during exposure and precultivation [mg], *n*_pot_ and *n*_seed_ are the total N content [mg] of the soil–plant model system (i.e., substrate, solution, plant) after exposure and the seeds used per system, respectively, and 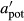 and 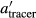 represent the excess ^15^N abundance [at. %] (determined ^15^N abundance minus natural ^15^N abundance of 0.3663 at. %) of 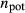 and the applied tracer, respectively.

The ^15^N recovery was calculated as the following:

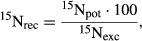
2

where ^15^N_rec_ is the ^15^N recovery [%], ^15^N_pot_ the excess ^15^N abundance in the pots [mg], and ^15^N_exc_ the excess ^15^N abundance in applied fertilizer [mg].

Calculation of total N recovery was performed according to:

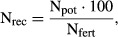
3

where N_rec_ is the total N recovery [%], N_pot_ the total N content in pots at the end of the experiments [mg], and N_fert_ the N content of grass seedlings and applied fertilizer [mg].

### Detection of ^15^N_2_O and ^15^N_2_ in substrate and nutrient solution

Concentrations of ^15^N_2_O and ^15^N_2_ were determined in substrate and nutrient solution to detect possible gaseous N losses via denitrification. Substrate air samples were taken from bottom (*n* = 4) and middle (*n* = 4) parts of the vegetation pots in spring 2013 after exposing pots in the field for 1 year. The sampling was performed using a custom-built cannula (length 200 mm; diameter 1.5 mm) connected to a syringe (20 mL, Terumo Europe N.V., Leuven, Belgium) and an evacuated Exetainer® (12 mL, Labco Limited, High Wycombe, Buckinghamshire, UK) by a three-way valve (Discofix, Braun Melsungen AG, Melsungen, Germany). The Exetainer® was filled with 15 mL soil air. Nutrient solution samples were taken using a syringe (50 mL, Omnifix, Braun Melsungen AG, Melsungen, Germany) connected to a glass vial (120 mL). Glass vials were flushed with helium prior to usage and 60 mL were evacuated. Soil air and headspace ^15^N_2_ and ^15^N_2_O samples were measured immediately after sampling using a Stable Isotope Ratio Mass Spectrometer (MAT 253, Thermo Scientific, Bremen, Germany). The detection limit (7.45 ppm) was defined as two times the standard deviation of the external reference gas (air-N_2_, *n* = 12).

A rough estimate of the minimum detectable flux can be obtained using Fick’s 2^nd^ law of diffusion:

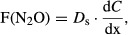
4

where F(N_2_O) is the N_2_O flux, *D*_s_ the diffusivity in soil [cm^2^ s^−1^], and d*C*/dx the N_2_O concentration gradient. A best estimate of *D*_s_ is obtained using the empirical approach proposed by Millington and Quirk (1961):

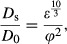
5

where *D*_s_/*D*_0_ is the relative diffusivity, *D*_0_ the diffusivity in air [cm^2^ s^−1^], *ε* the volumetric air content [cm^3^ s^−3^], and *φ* the porosity [cm^3^ cm^−3^]. Assuming a bulk density of 1.5 and volumetric water content of 0.2 L L^−1^, we obtain a diffusion coefficient of 0.0085 cm^2^ sec^−1^. The water content is estimated assuming a tension of −10 hPa at the middle part of the pot as there was free outflow of water at the bottom of the pot and thus suggesting zero tension using a default soil water retention curve for sandy soil (Durner and Flühler 2005).

### Extrapolation from pot to field scale and from the growing season to 1 year

As the ITNI approach is a pot-based experiment, extrapolation to the field scale is necessary. N deposition is expected to be controlled by either plant species, the physiological status, shape of the plant, and/or development of dry matter (Russow and Böhme 2005), which basically gives three ways to extrapolate data to the field scale: extrapolation based on (1) pot area, (2) aboveground dry matter, and (3) a combination of both. Previous studies using an ITNI setup almost exclusively extrapolated data based on the area method (e.g., Böhlmann et al. 2005). Potential problems in extrapolating results from pot to field scale were discussed by Russow and Böhme (2005). In this study, extrapolation of data to field scale was performed based on the pot area.

To compare plant biomass of natural vegetation and of our monitor plants, we sampled aboveground biomass from three plots (1 × 1 m) in the field in July 2014 comprising (1) mainly bog grasses *Eriophorum vaginatum* and *Molinia caerulea*, (2) mainly *Erica tetralix* and *Sphagnum* mosses, and (3) a mix of bog grasses, bog heather, and mosses. Biomass samples were dried for 96 h at 60°C in a compartment drier. Field biomass samples were used to exemplarily extrapolate data based on aboveground dry matter.

As ITNI experiments are usually carried out only during the growing season to avoid plant senescence and N losses due to outgassing of NH_3_ during winter, data have to be extrapolated to 1 year in order to quantify annual N deposition. However, physiological inactivity of plants during winter has to be considered, and extrapolation of results obtained during the growing season might lead to an overestimation of annual atmospheric N deposition. To evaluate potential uncertainties in annual airborne N input extrapolated from data, we carried out ITNI experiments over an experimental period of 1 year using both perennial and annual grass species.

### Statistical evaluation of data

R studio (version 3.1.0; R Core Team, 2012) was used to perform a Student’s t-test for analyzing the relationships between properties of the two monitor plants *Eriophorum vaginatum* and *Lolium multiflorum* (e.g., total vs. aboveground biomass production, share of total biomass on atmospheric N uptake vs. share of substrate on airborne N deposition). All tests were based on a 95% confidence interval and results are given as significant (*P *<* *0.05).

## Results

### Mass of aboveground biomass in the field

Biomass sampling in the field resulted in aboveground dry matter of 576.5 ± 28.8, 517.0 ± 25.9, and 303.3 ± 15.2 g m^−2^ for the bog grass dominated plot, the bog heather and S*phagnum* moss dominated plot, and the plot with mixed bog grasses, bog heather and mosses, respectively. Based on these results, grass biomass has a share of slightly above 50% of total biomass in the field, and we assume average field aboveground grass biomass to be in the order of 300 g m^−2^.

### Monitor plant *Lolium multiflorum*

The produced above- and belowground biomass and total atmospheric N deposition determined with the ITNI system using *Lolium multiflorum* in the first year of investigation are shown in Table1. Four of five vegetation pots showed a similar aboveground biomass production with a maximum variability of 5%. Only one biomonitor produced about 30% less biomass than the other pots. The share of roots to total biomass was with an average of about 70% significantly higher than the produced aboveground biomass (*P *<* *0.05).

**Table 1:** Dry matter (DM) and content of total and airborne N in different compartments of the biomonitor system and calculated mean daily allocation rates of deposited N for the biomonitor experiment with *Lolium multiflorum* in the first year of investigation (±standard deviation (SD))

Fraction	Mass/Volume (g DM pot^−1^; mL pot^−1^)	N content (mg g^−1^ DM; mg mL^−1^)	Deposited N (mg g^−1^ DM; mg mL^−1^)	Deposited N (mg pot^−1^)	N allocation rate (*μ*g d^−1^ pot^−1^)
Aboveground plant	8.5 ± 1.3^1^	17.9 ± 2.0	1.7 ± 0.2	14.3 ± 1.8	107.9 ± 6.3
Roots	23.2 ± 10.9^1^	6.0 ± 1.7	0.4 ± 5 × 10^−2^	8.4 ± 3.5	63.4 ± 23.3
Substrate	10000.0 ± 0.0^1^	3 × 10^−3^ ± 3 × 10^−4^	9 × 10^−4^ ± 1 × 10^−4^	9.3 ± 1.2	71.0 ± 13.8
Nutrient solution	1997.9 ± 284.0^2^	2 × 10^−3^ ± 1 × 10^−3^	5 × 10^−4^ ± 2 × 10^−4^	1.1 ± 0.7	9.2 ± 7.4
Whole system	12029.7 ± 284.3	–	–	33.2 ± 5.1	251.7 ± 30.5

1 Mass of fraction.

2 Volume of fraction.

The average amount of atmospheric N deposited into a single monitor system during the whole experimental period (precultivation and exposure in the field) is calculated from total N contents and ^15^N signatures of the different monitor components (aboveground biomass, roots, substrate, and nutrient solution) and was 33.2 ± 5.1 mg N pot^−1^ (Table1). Total N deposition amounted to about 7% of initially total added N with fertilizer. The different exposure times (differences up to 30 days) of pots in the field have to be considered when comparing average airborne N deposition to the single biomonitor systems. The mean allocation rate per pot and day, however, allows for the direct comparison between the different vegetation pots and was on average 251.7 ± 30.5 *μ*g N d^−1^ pot^−1^ (Table1).

A considerably higher contribution of about 70% of total deposited airborne N was found for plant biomass, while the rest (30%) was allocated to substrate and nutrient solution. Previous studies showed that the variability in N allocation rates between the single vegetation pots was larger than the methodological error during sample preparation and chemical analyzes (Russow and Weigel 2000) and was on average 12% for our experiments. Highest variabilities between pots of up to 47% were found for root samples from the different vegetation pots.

**Figure 2:**
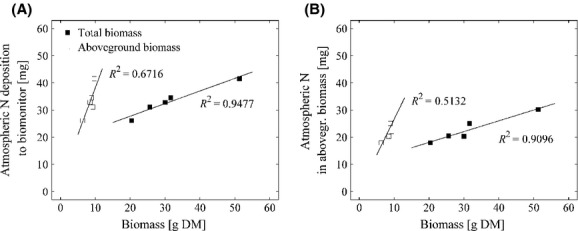
Relation between airborne N in the biomonitor system and produced biomass for aboveground and total biomass (A) and dependence between atmospheric N in aboveground parts of the monitor plant and biomass production of aboveground and total biomass (B). Results from the experimental year 2011.

The dependence between atmospheric N deposition and produced biomass for the single biomonitor systems is shown in Figure2. Airborne N in both aboveground and total biomass increased with increasing mass of aboveground and total biomass.

**Figure 3:**
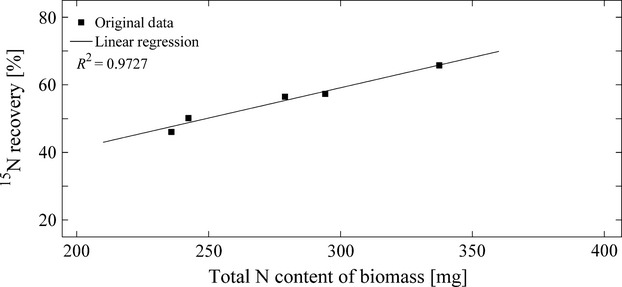
Dependency between total N contents of biomass and ^15^N recoveries of *Lolium multiflorum* in 2011.

Total N recoveries ranged from 52.6 to 74.0% with an average of 61.8% and the mean excess ^15^N recovery was 55.2% varying from 46.1 to 65.8% (cf. Table S1). As shown in Figure3, a positive linear relationship between total N contents of total produced biomass and excess ^15^N recoveries (*R*^2^* *=* *0.97) could be found. Total N contents of substrate and nutrient solution did not show a clear dependency on excess ^15^N recoveries (data not shown).

**Figure 4:**
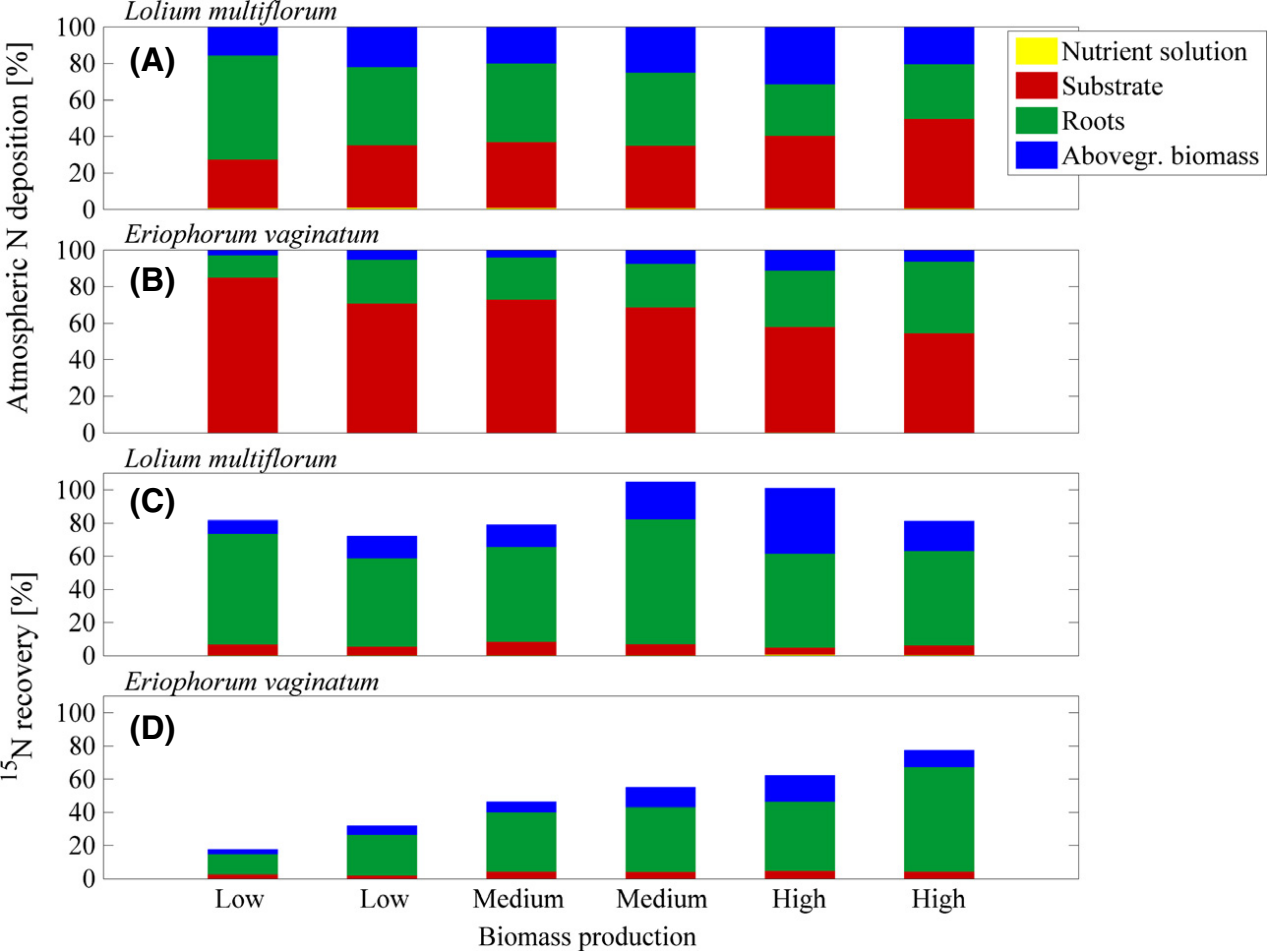
Proportion of total atmospheric N deposition (A and B) and contribution of ^15^N excess (C and D) found in different compartments of the biomonitor systems to the total ^15^N recovery in dependency to level of total biomass production during experiments in 2012.

The experiments using *Lolium multiflorum* under the same experimental conditions during the second year of investigation (N application 0.5 g pot^−1^) showed an average total and aboveground biomass production of 31.2 ± 3.1 g DM and 8.7 ± 0.9 g DM, respectively, which was in the same range as the biomass production during the first year (cf. Table1). The mean atmospheric N deposition to the biomonitor system resulted in 570.1 ± 40.3 *μ*g N d^−1 ^pot^−1^. About 55% of total atmospheric N deposition was allocated to plant biomass; substrate and nutrient solution contributed to total N uptake with on average 45% (high level of total biomass production, cf. Fig.4). The total N and excess ^15^N recoveries were on average 111% and 91%, respectively.

Of all investigated pots, the share of roots on total biomass production amounted on average to 77.8% ranging from 66.8 to 83.6% and was thus significantly higher than aboveground biomass production (*P *<* *0.05).

**Figure 5:**
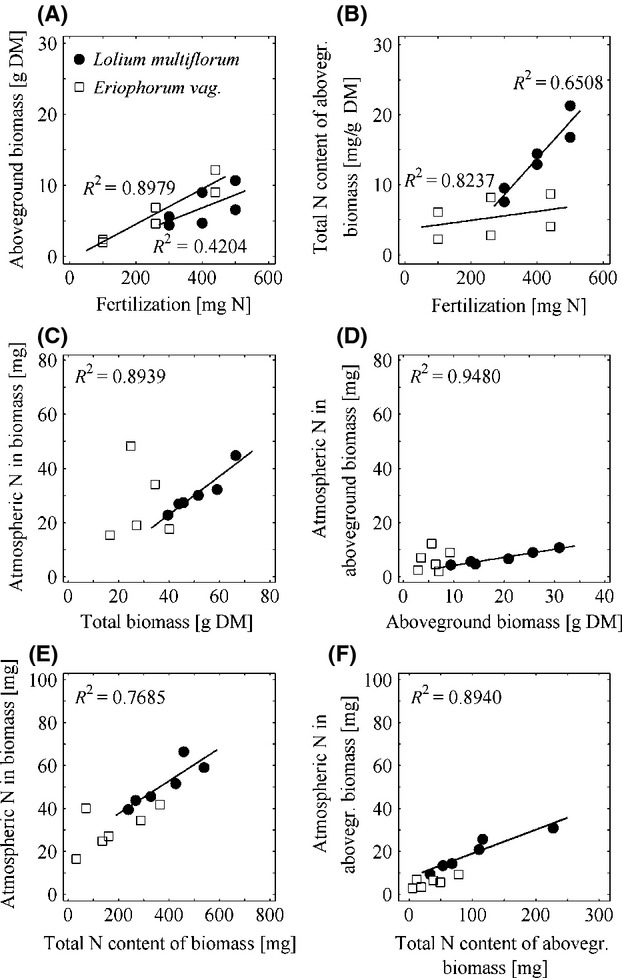
Dependence between fertilization level and produced aboveground biomass and total N content of aboveground biomass (A and B), atmospheric N in total biomass and aboveground biomass in relation to total biomass and aboveground biomass (C and D), and relations between atmospheric N in biomass and total N content of biomass (E) and atmospheric N in aboveground biomass and total N content of aboveground biomass (F) for both *Lolium multiflorum* and *Eriophorum vaginatum* in 2012.

In general, a positive dependency between applied N fertilizer and the development of aboveground biomass (Fig.5A) and total N contents of aboveground biomass (Fig.5B) could be observed. Furthermore, atmospheric N in both total biomass and aboveground biomass was found to be positively correlated to the produced total and aboveground biomass (Fig.5C, 5D) and to the total N contents of total and aboveground biomass (Fig.5E, F).

On average, 64 and 68% of total atmospheric N deposition was allocated to plant biomass under medium and low total biomass production, respectively (cf. Fig.4). Substrate and nutrient solution contributed to total N uptake with on average 36 and 32% (medium and low level of total biomass production, cf. Fig.4). The mean atmospheric N deposition to the biomonitor system resulted in 495.2 ± 49.5 *μ*g N d^−1^ pot^−1^ (moderate biomass production) and 337.9 ± 33.8 *μ*g N d^−1^ pot^−1^ (low biomass production), respectively. As shown in Figure4, the partitioning of airborne N within the biomonitor system differed with increasing biomass production. While the relative proportion of total airborne N in roots decreased with increasing total biomass production, atmospheric N in aboveground biomass positively correlated with the produced amount of aboveground and total biomass (cf. Fig.4A, 5C, D).

**Figure 6:**
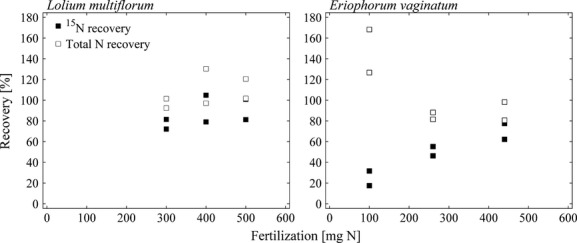
Excess ^15^N and total N recoveries in relation to N added with fertilizer for both *Lolium multiflorum* and *Eriophorum vaginatum* in 2012.

Total N and excess ^15^N recoveries of all six vegetation pots were on average 107.2 and 86.6% varying from 92.4 to 130.3% and from 72.1 to 104.8%, respectively (Table S2). As shown in Figure6, total N and excess ^15^N recoveries increased with increasing addition of ^15^N fertilizer. Furthermore, the highest share of ^15^N excess was found in biomass, whereas no dependency between biomass production and the relative share of fractions on total ^15^N excess could be observed (Fig.4C).

Furthermore, in correspondence with findings for *Lolium multiflorum in* 2011 (cf. Fig.3), total N contents of biomass and excess ^15^N recoveries positively correlated (*R*^2^* *=* *0.71), while no such relationship could be found for substrate and nutrient solution (data not shown).

### Monitor plant Eriophorum vaginatum

Using *Eriophorum vaginatum* as monitor plant during the second year of investigation, total biomass production ranged from 15.3 to 89.6 g DM. The share of roots on total biomass was only slightly higher as observed during experiments with *Lolium multiflorum* and varied from 73.6 to 88.6% with an average contribution of 82.4%. Root biomass production was significantly higher than the produced amount of aboveground biomass (*P *<* *0.05). No significant difference could be found between root production of *Eriophorum vaginatum* and *Lolium multiflorum* in 2012 (*P *>* *0.05).

As shown in Figure5A, aboveground biomass production increased with increasing fertilization. Furthermore, increasing fertilization of monitor plants led to an increasing N status of aboveground biomass (cf. Fig.5B). During the whole experimental period including precultivation and exposure in the field, the atmospheric N deposition ranged from 81.9 to 138.2 mg N pot^−1^. The mean allocation rate per pot and day was in the range from 342.9 to 580.6 *μ*g N d^−1^ pot^−1^. While the atmospheric N in both aboveground and total biomass increased with total N contents of aboveground and total biomass (cf. Fig.5E, F), no distinct dependency could be found for atmospheric N in total and aboveground biomass in relation to the produced total and aboveground biomass (cf. Fig.5C, D).

In contrast to the experiments using *Lolium multiflorum* as monitor plant, most of the deposited atmospheric N was found in the substrate (up to 85%) (cf. Fig.4A, B). While vegetation pots with high biomass production resulted in a share of on average about 44% of total biomass on airborne N uptake, the contribution of biomass decreased with decreasing amount of produced biomass (cf. Fig.4b).

While no dependency between total N contents of substrate and nutrient solution and excess ^15^N recoveries could be observed, a strong positive linear relationship between total N contents of biomass and excess ^15^N recoveries was found (*R*^2^* *=* *0.92, data not shown).

Total N and excess ^15^N recoveries of all six vegetation pots were on average 107.2 and 48.4% ranging from 80.6 to 168.2% and from 17.5 to 77.5%, respectively (Table S3). While excess ^15^N recoveries positively correlated with initially added ^15^N with fertilizer, total N recoveries decreased with increasing N fertilization of vegetation pots and biomass production (cf. Fig.6). A positive dependency between produced biomass and share of biomass on ^15^N excess could be observed (Fig.4D). In correspondence with findings for *Lolium multiflorum*, highest shares of ^15^N excess were found in biomass.

### ^15^N_2_O and ^15^N enrichment in substrate and nutrient solution

The maximum concentration of fertilizer-derived N_2_ and N_2_O in soil air samples taken from the middle part of the pot was 3.7 ppm and was thus below the detection limit. Still, flux calculation resulted in 45 mg N pot^−1^ yr^−1^, that is, about 15% of the N added with fertilizer and gives thus a rough estimation of the minimum detectable flux.

Mean fertilizer-derived N_2_ and N_2_O in the headspace samples of the nutrient solution was 14.8 ppm and was hence above the detection limit. This concentration corresponded to 3.8 *μ*g N per solution container assuming a concentration of dissolved air N_2_ according to N_2_ solubility at 10°C (Weiss 1970). Extrapolating this number to the entire period results in 111.1 *μ*g N and thus explains only a small part of N losses. However, it is a direct proof of denitrification in the experimental setup. We suspect that these dissolved ^15^N gases were leached from the vegetation pot and thus probably indicate denitrification in the rooting zone of the plants.

### Year-round experiments

During the second year of experiments, two pots per grass species remained exposed in the moorland during winter time (397 days). This year-round experiment resulted in N uptake ranging from 216.7 to 524.0 *μ*g N d^−1^ pot^−1^ depending on grass species (Table2). Both *Eriophorum vaginatum* and *Lolium multiflorum* showed a positive correlation between atmospheric N deposition and produced biomass. In contrast to experiments carried out during the growing season, biomass production decreased with increasing N fertilization. Total N and excess ^15^N recoveries ranged from 58.5 and 40.1% to 125.1 and 72.9% (*Lolium multiflorum*) and from 88.0 and 37.0% to 286.1 and 53.6% (*Eriophorum vaginatum*), respectively.

**Table 2:** N supply, total biomass production, N content of total biomass, N deposition to monitor systems, and N allocation rates of vegetation pots (*n* = 2) exposed in the field for 1 year

Species	N supply (mg)	Total biomass (g DM pot^−1^)	N content (mg g^−1^ total DM)	Deposited N (mg g^−1^ total DM)	Deposited N (mg pot^−1^)	N allocation rate (*μ*g d^−1^ pot^−1^)
*Lolium multiflorum*	300.0	16.0	13.3	4.2	157.1	368.8
	500.0	11.2	14.0	3.8	92.3	216.7
*Eriophorum vaginatum*	100.0	36.9	5.0	4.3	252.0	524.0
	440.0	25.8	8.4	2.3	152.1	316.3

## Discussion

### Monitor plant *Lolium multiflorum*

On average, 55% of initially added ^15^N tracer was recovered in the ITNI system during the first year of investigation. In contrast to the results from experiments carried out in 2012, this indicates substantial losses of ^15^N in the first year. The high ^15^N losses might have occurred during the growing season (e.g., losses of biomass, nutrient solution) and during extraction of samples (e.g., harvest of biomass, homogenization of samples). Based on the findings from the detection of ^15^N_2_O and ^15^N_2_ in nutrient solution and soil air, small losses of N from solution cannot be excluded. The strong linear dependency found between total N contents of total biomass and excess ^15^N recoveries may indicate that N losses mainly occurred from plant biomass. However, as we did not investigate NO emissions, nitrification processes might enhance potential gaseous N losses from substrate and their actual significance may be greater. As stated by Russow and Weigel (2000), a ^15^N recovery below 80% has to be critically regarded with respect to the calculated total atmospheric N input. A ^15^N recovery of <100% will always result in an underestimation of the exact airborne N input, whereby the magnitude of discrepancy depends on the type and time of the ^15^N losses (e.g., denitrification, loss of ^15^N-labeled leaves, loss of ^15^N-labeled solution during harvesting). In any case, however, underestimation cannot fall below the ^15^N recovery rate, and hence, calculated atmospheric N input in the present experiment corresponds on average to at least 55% of the “real” airborne N input. Due to the high share of about 85% of biomass on total N contents of biomonitors, excess ^15^N recoveries >100% most likely resulted from measurement uncertainties when determining N contents and ^15^N contents of plant biomass samples. The positive dependency between total N contents of biomass and excess ^15^N recoveries and the high share of grass biomass on ^15^N excess (Fig.4C) show that a low excess ^15^N recovery of experiments is most likely related to plant N losses during, for example, exposure of vegetation pots in the field, sample preparation, or measurement uncertainties of N determination in biomass samples using isotope ratio mass spectrometry coupled to an elemental analyzer.

A high dependency of plant biomass on atmospheric N uptake has been also shown by He et al. (2007) using *Zea mays* and *Lolium multiflorum* as monitor plant. Using *Lolium perenne* in pot experiments at three different levels of N fertilization ranging from 300 to 900 mg N, Böhme et al. (2003) also found increasing biomass production and subsequent increasing N deposition: About 760 *μ*g N d^−1^ pot^−1^ was deposited to pots with high biomass production (127.29 ± 5.76 g DM), while at medium (83.75 ± 1.37 g DM) and low (44.45 ± 8.71 g DM) levels of biomass production only about 610 and 590 *μ*g N d^−1^ pot^−1^ could be found. It is thus suggested that increasing airborne N uptake with increasing biomass in the pots most likely resulted from larger leaf areas of the grasses and thus higher N uptake through stomata and cuticles.

On average, the total amount of atmospheric N was significantly higher in shoots than roots of our monitor plants (*P *<* *0.05). Furthermore, atmospheric N found in total biomass was significantly higher than in substrate (*P *<* *0.05). Based on the fact that the share of aboveground biomass on total atmospheric N uptake positively correlated with total and aboveground biomass production, it can be assumed that the main entry path for airborne N changed from substrate (wet N deposition) to aboveground biomass (N uptake through stomata and cuticles) most likely due to the lower wet N deposition to the sand surface as the coverage of the substrate increases. In contrast, it has been previously reported that dependent on location and used monitor plant, the main entry path for atmospheric N is the soil substrate by wet N deposition to the substrate cover. Böhlmann et al. (2005) as well as Russow and Weigel (2000) found highest N deposition to sand and nutrient solution, while N uptake by *Calamagrostis villosa* and different types of grain accounted for 9–11% to 32% of total atmospheric N deposition, respectively. Böhlmann et al. (2005) report low biomass production to be the main factor causing this relatively low contribution of plant uptake on total atmospheric N deposition.

Due to the similar developmental stage of plants during experiments with same amounts of added N fertilizer in both 2011 and 2012 (500 mg N), the considerably higher calculated atmospheric N uptake in 2012 compared to 2011 most likely resulted from higher average excess ^15^N recovery rates of 91%. Furthermore, the vegetation pots were already exposed in the field in May 2012, thus taking into account N deposition peaks due to N emissions from fertilization of surrounding arable land in late spring 2012 (Hurkuck et al. 2014). As shown in that study, year-to-year variability in total N deposition of 0.4 kg N ha^−1^ yr^−1^ to the site determined with a micrometeorological approach (KAPS-denuder-filter systems) and bulk samplers was low during two consecutive experimental periods. However, it has to be taken into account that this approach disregards species-specific plant N uptake and might therefore underestimate total N deposition as determined by ITNI. The deviation found in N input into the biomonitor between both years is therefore most likely not caused by differing N depositions within this region but can be attributed to methodological shortcomings (low excess ^15^N recovery) during the first year of investigation.

### Monitor plant Eriophorum vaginatum

The missing dependency of atmospheric N deposition on biomass production was in contradiction to the observations during experiments using *Lolium multiflorum* as monitor plant and differed also from previous studies (Russow and Weigel 2000; Leith et al. 2001; Böhlmann et al. 2005; Russow and Böhme 2005; He et al. 2007). In contrast to *Lolium multiflorum*, *Eriophorum vaginatum* showed a less extensive plant growth within the vegetation pots resulting in a lower coverage of the substrate surface. It can thus be assumed that the main atmospheric N input happened by dry and wet N deposition on the substrate with following N uptake from substrate by roots and transport of assimilated 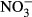 and 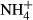 to the plant leaves and shoots.

This is further confirmed by the high share of the substrate on atmospheric N uptake which decreased with increasing biomass production and subsequent increasing substrate coverage. The amount of airborne N was larger in substrate than in the monitor plant (*P *<* *0.05). In comparison with the experiments using *Lolium multiflorum* as monitor plant in 2012, atmospheric N found in the substrate was significantly higher (*P *<* *0.05).

The strong positive correlation between total N contents of biomass and excess ^15^N recoveries as well as the high share of biomass on ^15^N excess of the biomonitor (Fig.4D) indicates that low excess ^15^N recoveries of experiments mainly occurred due to ^15^N losses from biomass.

While total N recoveries ranged from slight losses of N (up to 20%) and moderate N uptake (up to 50%), excess ^15^N recoveries during experiments with low biomass production (17.5%) indicated substantial N losses from vegetation pots during, for example, exposure of pots in the field, sample preparation, and uncertainties in ^15^N analysis. As described above, this may result in a strong underestimation of the exact airborne N input (cf. Section Monitor plant *Lolium multiflorum*) and the “real” airborne N deposition might be up to about fivefold higher. As discussed by Russow and Böhme (2005), increasing N fertilization may not only lead to an increasing biomass production and subsequent increasing N uptake but might also limit N uptake due to the enhanced mesophyll resistance caused by increasing apoplastic N content (Frank and Marek 1983; Rowland et al. 1985).

### Extrapolation of data from the growing season and year-round experiments

Data from ITNI experiments carried out only during the growing season have to be extrapolated to determine annual N input. As discussed elsewhere (Russow and Böhme 2005), extrapolation of data is subjected to uncertainties which mainly depend on the applied method and how representative the used monitor plant is compared to the natural field vegetation. In correspondence with most of the previous ITNI studies, extrapolation of data was carried out based on the pot area, assuming deposition rates during non-growing season to be similar as during growing season as high water table, rain, and fog in winter time result in high N deposition via nonstomatal pathways which may thus compensate for the missing plant uptake.

**Figure 7:**
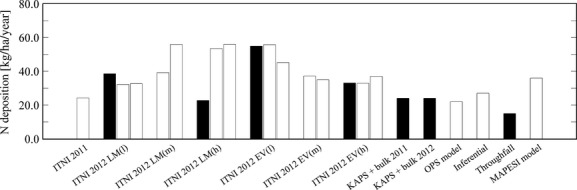
Overview of results from different methods to determine atmospheric N input into the study site (black bars indicate year-round experiments, LM = *Lolium multiflorum*, EV = *Eriophorum vaginatum*, l = low, m = medium and h = high biomass production).

Calculation of total airborne N input into pots planted with *Lolium multiflorum* and high N status amounts to 24.2 ± 2.9 kg N ha^−1^ yr^−1^ for the first experimental period (Fig.7). Extrapolation of data from experiments with low, medium, and high biomass production during the growing season resulted in 32.5 ± 3.2 kg N ha^−1^ yr^−1^, 47.6 ± 4.8 kg N ha^−1^ yr^−1^, 54.8 ± 5.5 kg N ha^−1^ yr^−1^ (*Lolium multiflorum*) and 50.5 ± 5.1 kg N ha^−1^ yr^−1^, 36.1 ± 3.6 kg N ha^−1^ yr^−1^, 34.9 ± 3.5 kg N ha^−1^ yr^−1^ (*Eriophorum vaginatum*), respectively (Fig.7).

We additionally compared results from extrapolation of pots exposed during the growing season and year-round experiments with low and high biomass production (Fig.7). In general, year-round ITNI experiments corresponded well with extrapolated data. Only the year-long growth and high biomass production of *Lolium multiflorum* resulted in substantially lower annual N deposition which was most likely due to the low excess ^15^N recovery of 40%.

### Comparison to other studies

As part of the ERNST project (Landkreis Emsland, Germany, 2013), different methods have been applied to determine and model N loads into the study site which are described in the below-mentioned references: throughfall method, micrometeorological approach (KAPS-denuder-filter systems; Peake 1985; Hurkuck et al. 2014) complemented with bulk samplers, inferential modeling, “Operational Priority Substances” (OPS) modeling (Van Jaarsveld 2004), and the ITNI experiments presented here (Fig.7). Figure7 presents an overview of the obtained results and further compares them to the total N deposition into the investigated area calculated by the German Environmental Agency using the MAPESI model (Modelling of Air Pollutants and EcoSystem Impact, Gauger et al. 2008).

On average, atmospheric N deposition was highest when determined by ITNI experiments (Fig.7). This confirms the previous assumption that ITNI additionally considers plant species-specific N uptake and thus may generally result in higher N input compared to N deposition modeling based on the big leaf concept. However, the low excess ^15^N recovery during some experiments resulted in a relatively high uncertainty and the actual airborne N input into the biomonitors may be strongly underestimated. Considering the excess ^15^N recovery rate of on average 55% in 2011 and during experiments with vegetation pots exposed for a whole year, the actual N input accounts for at least the determined input and at maximum a possible N uptake of twice as high as the determined input (i.e., 48 (*Lolium multiflorum* year 1) to 110 kg N ha^−1^ yr^−1^ (*Lolium multiflorum* year 2), respectively).

A previous study by Böhlmann et al. (2005) using *Calamagrostis villosa* as monitor plant within a mire in the *Hochharz Mountains* in Germany resulted in a mean atmospheric N deposition of 30 kg N ha^−1^ yr^−1^ (extrapolation of data based on the pot area), which differed only little from wet-deposited N. The biomass developed poorly and the study area was not in close vicinity to agriculturally used land. The authors explain the increased N input by N capture through surrounding forest canopy. Using *Zea mays*, *Hordeum vulgare L*., and *Secale cereale* atmospheric N deposition into a study site in Central Germany ranged from 45 to 75 kg N ha^−1^ yr^−1^ (Böhme et al. 2002). Extraordinary high N deposition of up to 117 kg N ha^−1^ yr^−1^ using *Zea mays* was found in the North China Plain, one of the most intensively used agricultural regions in China (He et al. 2010b). All studies conducted on this subject found a dependency between the total atmospheric N deposition and the season, the used monitor plant as well as the development of biomass (Böhme et al. 2002; Böhlmann et al. 2005; Russow and Böhme 2005; He et al. 2007, 2010b).

In the present study, the determined total airborne N input in both years exceeded the defined ecosystem-specific critical load of 5–10 kg N ha^−1^ yr^−1^ at least two- to fivefold. Thus, the ecosystem might be subjected to an increased sensitivity and shifts in plant species compositions cannot be excluded (cf. Section Uncertainties of ITNI experiments).

### Potential ecosystem response to increasing N deposition

In general, increased N deposition has been shown to substantially affect moorland vegetation: Steubing and Buchwald (1989) and Heil and Diemont (1983) describe a transition of heathlands to grasslands in northern Germany and the Netherlands. Bobbink et al. (2010) reported N saturation in *Sphagnum* mosses with significant changes in *Sphagnum* cover of mire, bog, and fen habitats. Tomassen et al. (2003) found stimulation of growth of *Molinia caerulea* and *Betula pubescens*, more nitrophilous grass and tree species, in an ombrotrophic bog in the Netherlands. To evaluate ecological effects of increased atmospheric N input, the uptake pathway and form of dominantly available N species have been taken into account. According to Van der Eerden et al. (1990), NH_3_ uptake by foliar is more rapid than 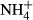 and 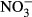 assimilation by roots and foliar. Furthermore, Dueck et al. (1991) reported that NH_3_ is mainly taken up through plant stomata instead of cuticles. As a consequence, N deposition in the form of gaseous NH_3_ was found to have greater effects on moorland vegetation than wet-deposited 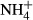 (Leith et al. 2002). They found increasing shoot extension after the exposure of *Calluna vulgaris* to increasing doses of dry NH_3_ (2–90 *μ*g m^−3^). As shown by Hurkuck et al. (2014), more than 80% of total dry-deposited N to the here-presented study site resulted from NH_3_ deposition. It can thus be assumed that NH_3_ is the N compound mostly contributing to N uptake. Van der Eerden (1982) observed different crop plants (e.g., *Lolium multiflorum* cv. Optima) exposed to increased NH_3_ concentration levels. They reported both direct and indirect effects, such as necrosis, growth reduction, NH_3_-specific symptoms (e.g., black spots on leaves), and increased sensitivity to cold, respectively, as major responses of plants to NH_3_ fumigation. *Lolium multiflorum* (cv. Optima) did not show foliar injury during their experiments and it was suggested that crop grasses (e.g., *Lolium multiflorum* LAM. cv. RVP) are insensitive to increased NH_3_ concentrations of up to 709 *μ*g m^−3^ (Whitehead and Lockyer 1987). However, these studies have been carried out over a maximum period of 33 days. Long-term field observations using ombrotrophic mire-specific vegetation (e.g., *Calluna vulgaris*, *Eriophorum vaginatum*) exposed to increasing levels of NH_3_ and 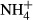 supply over 2 years have been carried out by Leith et al. (2002). There was no species loss or foliar injury during their experiments, and they suggested that only the presence of secondary stress factors (e.g., drought) initiates changes in species composition.

In the present study, we did not observe plant responses other than changes in biomass production to increased N fertilization during our experiments. The plants were exposed in the field for a period of 1 year at maximum, and as has been previously shown, we assume that effects on increased N deposition occur only during long-term investigations and under the presence of secondary stress factors. Furthermore, maximum NH_3_ concentration at the study site integrated over 1-week periods was about 15 *μ*g m^−3^ and was thus comparably lower than those in the above-mentioned experiments (Hurkuck et al. 2014). The measurement site had a distance of at least 2 km to the next farm, and there were thus no local effects of the strongest emitters and ambient air N concentrations. Still, the high share of NH_3_ on total N input might lead to an increased sensitivity of ombrotrophic vegetation to, for example, frost, growth reduction, and toxic effects such as necrosis when atmospheric N deposition increases. Consequently, a change in the biodiversity of the ecosystem could promote the drainage of the bog and a subsequent alteration of the local hydrological regime.

### Uncertainties of ITNI experiments

Precultivation of grasses was carried out in a glasshouse, and atmospheric N uptake by plants prior to their exposure in the field could not be excluded. However, the overestimation of airborne N uptake during the exposure at the study site due to atmospheric N deposition during precultivation is expected to be low as plants were protected from external influences and the period of precultivation was kept as short as possible (on average 50 days, ranging from 14 to 83 days).

In comparison with other ITNI studies (Russow and Weigel 2000; : 80%, Weigel et al. 2000: 80–99%), the experiments using *Lolium multiflorum* in 2011 and *Eriophorum vaginatum* in 2012 showed a low ^15^N recovery. This has to be taken into account when estimating N input using the soil–plant model system, and atmospheric N uptake might be considerably higher. However, so far most studies on biomonitoring approaches did not indicate ^15^N recovery rates and evaluation of results is difficult. To get a more detailed picture on the main reasons for low ^15^N recovery, dynamics of ^15^N recovery should be assessed. Repeated ^15^N_2_ and ^15^N_2_O flux measurements could help to more precisely quantify N losses from denitrification processes within the nutrient solution containers and/or substrate in future studies. Furthermore, ^15^N recoveries of the different compartments of the biomonitor should be determined directly after ^15^N supply to estimate source of losses.

Another error might have occurred due to the fact that the monitor plants differed from the site-specific vegetation when using *Lolium multiflorum* as monitor plant. However, using native *Eriophorum vaginatum* during the second year of experiments overcame this shortcoming. In general, the used monitor plant should be as representative as possible compared to natural vegetation (e.g., soil coverage, N status) to quantify real atmospheric N depositions.

Furthermore, ITNI experiments are usually carried out during the growing season and extrapolation of data to a whole year is critical. The exposure of vegetation pots only during the growing season and thus optimum growth conditions may lead to a strong overestimation of results when extrapolated to a whole year. To avoid underrepresentation of winter time, year-round experiments are crucial. However, they have to be critically regarded due to the fact that the used grass species were inactive (*Eriophorum vaginatum*) during winter time or even died off (*Lolium multiflorum*). Subsequently, N losses due to NH_3_ outgassing during senescence of plants could presumably not be avoided. In order to quantify annual atmospheric N deposition, we assume that year-round experiments can only result in realistic annual N depositions if the monitor plant is physiologically active throughout the whole year. Although both approaches corresponded well and resulted in similar annual N input, they are subjected to the above-mentioned uncertainties. We therefore suggest a combined approach consisting of an ITNI setup used during the growing season and micrometeorological techniques (e.g., KAPS-denuder-filter systems, Hurkuck et al. 2014) during non-growing season when the application of ITNI experiments is constrained due to the above-mentioned shortcomings.

Another source of uncertainty is the extrapolation from pot to field scale. This issue has been intensively discussed by Russow and Böhme (2005). They suggest a combination of area and dry matter method. He et al. (2010a) applied both the area method and a plant number method for extrapolation of data using *Zea mays* as monitor plant. It was found that the plant density within the pot has to be as representative as possible to achieve realistic results. If single plants are used as biomonitors, a correction factor has to be applied when using the area method.

In order to evaluate the comparability of the used monitor plants within the vegetation pots and the native vegetation in the surrounding area, pot data were additionally extrapolated based on aboveground dry matter of field plots. Dry matter sampling in the field resulted in aboveground grass biomass of on average about 3000 kg DM ha^−1^, in comparison with that vegetation pots contained aboveground biomass ranging from 526.3 to 3210.5 kg DM ha^−1^ depending on the used monitor plant and fertilization level when extrapolated from pot scale to one hectare. Atmospheric N in aboveground biomass of vegetation pots ranged from 0.2 to 11.3 kg N ha^−1^. Taking into account aboveground biomass in the field, we calculated atmospheric N in aboveground grass matter ranging from 1.4 to 10.7 kg N ha^−1^. Extrapolation of data based on dry matter sampling shows that airborne N in aboveground biomass of the biomonitors is in the same range as the estimated atmospheric N uptake by aboveground grass biomass in the field. However, this method has to be critically regarded as the plots in the field not only consisted of grasses but also comprised heath vegetation and *Sphagnum* mosses. As the dry matter method presented here considers only grass biomass, additional N uptake from atmosphere by other species in the field was not taken into account and actual plant N uptake might be substantially higher. We suggest that the most realistic results can be obtained from the dry matter method when monitor plants are representative in terms of size and N status compared to the native vegetation in the field.

Using *Lolium multiflorum* and *Eriophorum vaginatum* grown in the glasshouse prior to the experiments during a maximum exposure of 1 year resulted in an investigation of young plants compared to the natural vegetation of the study site. He et al. (2010a) found a relationship between N uptake and different growth stages for *Zea mays,* and it can be assumed that plant age and subsequent biomass production might affect N uptake. Young plants are expected to have higher nutrient demands due to their vegetative growth and the results presented here might overestimate N input into the used soil–plant systems compared to the actual plant N uptake by native perennial vegetation. However, the lower density of grasses in the pots compared to the field most presumably leads to a lower atmospheric N uptake through the leaf surfaces and might thus offset this overestimation.

Finally, due to constraints in the experimental setup, only one to three replicates could be used per fertilization level during the second year of investigation. The experiments in 2011, however, showed a high variability in N uptake between the single vegetation pots and uncertainties in experiments with pots exposed at the study site for a whole year (2012) can be only estimated.

Although the ITNI method was found to be subjected to a number of uncertainties, it is a valuable approach to determine atmospheric N deposition. ITNI is the only known technique providing direct measurements of both dry and wet deposition as well as direct N uptake by plants, thus considering plant-specific characteristics such as biomass and N status. In order to improve its performance and reduce uncertainties related to ITNI, representative species should be chosen (e.g., similar biomass production), ^15^N recoveries need to be in the order of 80% at least and the method used for the extrapolation of data to field scale and a whole year should be selected according to the comparability between monitor plants and native vegetation.

## Conclusion

Airborne N input into soil–plant systems was found to be dependent on the monitor plant and was on average higher when using *Eriophorum vaginatum*. It could be shown that grass species respond differently to increasing N supply. Based on our findings, it can be assumed that *Lolium multiflorum* takes up N according to biomass production and leaf area as well as total N contents of biomass. In contrast, it was found that the slow-growing bog-specific grass adapted to nutrient-poor conditions *Eriophorum vaginatum* is more sensitive most likely due to an increasing compensation point resulting from N saturation within the leaves when N supply increases. In comparison with other approaches, ITNI revealed the highest atmospheric N deposition as it considers direct N uptake by plants. However, the low ^15^N recovery rate during some experiments indicated an underestimation of the applied ITNI approach and the “real” airborne N input may be significantly greater, but maximally almost twice as high when considering an average ^15^N recovery of 55%. The determined total airborne N input in both years exceeded the defined ecosystem-specific critical load of 5–10 kg N ha^−1^ yr^−1^ at least two- to fivefold. The high share of NH_3_ on total N input might lead to an increased sensitivity of ombrotrophic vegetation to, for example, frost, growth reduction, and toxic effects such as necrosis when atmospheric N deposition increases. Consequently, a change in the biodiversity of the ecosystem could promote the drainage of the bog and a subsequent alteration of the local hydrological regime.

## Supporting Information

**Table S1.** Duration of experiments, volume, N contents and N recoveries, ^15^N contents and ^15^N recoveries, and calculated N deposition for the single fractions of *Lolium multiflorum* in 2011 (exp. = experiments (pre-cultivation and exposition in the field)).

**Table S2.** Duration of experiments, volume, N contents and N recoveries, ^15^N contents and ^15^N recoveries, and calculated N deposition for the single fractions of *Lolium multiflorum* in 2012 (exp. = experiments (pre-cultivation and exposition in the field)).

**Table S3.** Duration of experiments, volume, N contents and N recoveries, ^15^N contents and ^15^N recoveries, and calculated N deposition for the single fractions of Eriophorum vaginatum in 2012 (exp. = experiments (pre-cultivation and exposition in the field)).
