# Supplementary material for: Effects of grass species and grass growth on atmospheric nitrogen deposition to a bog ecosystem surrounded by intensive agricultural land use
Source: Ecol Evol. 2015 Jun 3;5(13):2556–71. doi: 10.1002/ece3.1534 (PMC4523353; doi:10.1002/ece3.1534)
Supplement: Supplementary file 1 [file ece30005-2556-sd1.docx]

*Suppl. Tab. 1. Duration of experiments, volume, N contents and N recoveries, ^15^N contents and ^15^N recoveries, and calculated N deposition for the single fractions of Lolium multiflorum in 2011 (exp. = experiments (pre-cultivation and exposition in the field)).*

| Pot number and | Duration of exp. | Mass/Volume | N content | ^15^N | ^15^N excess | Deposited N | N allocation rate |
| --- | --- | --- | --- | --- | --- | --- | --- |
| fraction | [days] | [g DM pot^-1^; mL pot^-1^] | [mg] | [at. %] | [mg] | [mg pot^-1^] | [µg d^-1^ pot^-1^] |
| 1_Abovegr. biomass | - | 8.9* | 165.5 | 5.0 | 7.6 | 15.1 | 102.1 |
| 1_Roots | - | 22.7* | 128.7 | 5.0 | 6.0 | 9.9 | 66.7 |
| 1_Substrate | - | 10000.0* | 25.7 | 3.7 | 0.8 | 8.9 | 60.3 |
| 1_Nutrient solution | - | 1724.0^+^ | 1.4 | 3.3 | 0.0 | 0.5 | 3.7 |
| 1_Whole system | 148 | - | 321.2 | 5.3 | 14.5 | 34.5 | 233.0 |
| 1_Recovery [%] | - | - | 64.2 | - | 57.3 | - | - |
| 2_Abovegr. biomass | - | 9.3* | 158.6 | 5.0 | 7.3 | 14.8 | 100.2 |
| 2_Roots | - | 16.3* | 120.4 | 5.2 | 5.8 | 5.6 | 37.5 |
| 2_Substrate | - | 10000.0* | 26.9 | 3.7 | 0.9 | 9.4 | 63.6 |
| 2_Nutrient solution | - | 2262.5^+^ | 7.8 | 4.6 | 0.3 | 1.3 | 8.8 |
| 2_Whole system | 148 | - | 313.7 | 7.5 | 14.3 | 31.1 | 210.4 |
| 2_Recovery [%] | - | - | 62.7 | - | 56.5 | - | - |
| 3_Abovegr. biomass | - | 8.5* | 130.0 | 5.0 | 6.0 | 11.9 | 111.1 |
| 3_Roots | - | 21.5* | 105.9 | 5.0 | 4.9 | 8.4 | 78.7 |
| 3_Substrate | - | 10000.0* | 22.2 | 3.1 | 0.6 | 10.1 | 94.5 |
| 3_Nutrient solution | - | 2340.0^+^ | 5.2 | 3.1 | 0.1 | 2.3 | 21.9 |
| 3_Whole system | 107 | - | 263.3 | 6.9 | 11.7 | 32.8 | 306.6 |
| 3_Recovery [%] | - | - | 52.6 | - | 46.1 | - | - |
| 4_Abovegr. biomass | - | 9.7* | 175.0 | 5.0 | 8.0 | 16.5 | 111.8 |
| 4_Roots | - | 41.6* | 162.4 | 5.0 | 7.5 | 13.6 | 91.8 |
| 4_Substrate | - | 10000.0* | 31.6 | 3.7 | 1.1 | 10.6 | 71.6 |
| 4_Nutrient solution | - | 1780.0^+^ | 1.3 | 2.6 | 0.0 | 0.7 | 4.7 |
| 4_Whole system | 148 | - | 370.3 | 5.2 | 16.7 | 41.5 | 280.1 |
| 4_Recovery [%] | - | - | 74.0 | - | 65.8 | - | - |
| 5_Abovegr. biomass | - | 6.3* | 130.4 | 4.9 | 5.9 | 13.0 | 114.4 |
| 5_Roots | - | 14.1* | 11.9 | 5.2 | 5.4 | 4.8 | 42.1 |
| 5_Substrate | - | 10000.0* | 26.1 | 4.0 | 0.9 | 7.4 | 64.9 |
| 5_Nutrient solution | - | 1883.2^+^ | 8.6 | 4.9 | 0.4 | 0.8 | 6.9 |
| 5_Whole system | 114 | - | 277.0 | 8.1 | 12.7 | 26.1 | 228.7 |
| 5_Recovery [%] | - | - | 55.4 | - | 50.2 | - | - |

*Mass of fraction, ^+^Volume of fraction

^15^N content of fertilizer: 5.431 at. %, total N content of fertilizer: 500 mg N

^15^N content of grass seed: 0.370 at. %, total N content of grass seed: 0.036 mg N
