# Supplementary material for: Effects of grass species and grass growth on atmospheric nitrogen deposition to a bog ecosystem surrounded by intensive agricultural land use
Source: Ecol Evol. 2015 Jun 3;5(13):2556–71. doi: 10.1002/ece3.1534 (PMC4523353; doi:10.1002/ece3.1534)
Supplement: Supplementary file 2 [file ece30005-2556-sd2.docx]

*Suppl. Tab.2. Duration of experiments, volume, N contents and N recoveries, ^15^N contents and ^15^N recoveries, and calculated N deposition for the single fractions of Lolium multiflorum in 2012 (exp. = experiments (pre-cultivation and exposition in the field)).*

| Pot number and | Duration of exp. | Mass/Volume | N content | ^15^N | ^15^N excess | Deposited N | N allocation rate |
| --- | --- | --- | --- | --- | --- | --- | --- |
| fraction | [days] | [g DM pot^-1^; mL pot^-1^] | [mg] | [at. %] | [mg] | [mg pot^-1^] | [µg d^-1^ pot^-1^] |
| 1_Abovegr. biomass | - | 4.4* | 33.4 | 4.1 | 1.2 | 9.4 | 52.3 |
| 1_Roots | - | 22.5* | 234.5 | 4.8 | 10.3 | 34.3 | 190.8 |
| 1_Substrate | - | 9000.0* | 35.8 | 3.2 | 1.0 | 16.2 | 89.9 |
| 1_Nutrient solution | - | 1265.0^+^ | 1.4 | 3.9 | 0.0 | 0.4 | 2.2 |
| 1_Whole system | 180 | - | 305.0 | 4.5 | 12.6 | 60.4 | 335.7 |
| 1_Recovery [%] | - | - | 101.5 | - | 81.5 | - | - |
| 2_Abovegr. biomass | - | 5.6* | 53.5 | 4.2 | 2.1 | 13.4 | 74.5 |
| 2_Roots | - | 17.2* | 158.8 | 4.8 | 8.2 | 26.1 | 145.0 |
| 2_Substrate | - | 8700.0* | 36.8 | 2.6 | 0.8 | 21.1 | 117.3 |
| 2_Nutrient solution | - | 1195.0^+^ | 1.5 | 3.6 | 0.0 | 0.6 | 3.3 |
| 2_Whole system | 180 | - | 277.4 | 4.4 | 11.2 | 61.2 | 340.1 |
| 2_Recovery [%] | - | - | 92.4 | - | 72.1 | - | - |
| 3_Abovegr. biomass | - | 4.7* | 67.8 | 4.4 | 2.8 | 14.3 | 80.9 |
| 3_Roots | - | 22.7* | 260.0 | 4.9 | 11.8 | 31.3 | 176.7 |
| 3_Substrate | - | 8980.0* | 58.5 | 3.2 | 1.7 | 26.0 | 147.0 |
| 3_Nutrient solution | - | 1260.0^+^ | 2.1 | 4.0 | 0.1 | 0.6 | 3.5 |
| 3_Whole system | 177 | - | 388.4 | 4.6 | 16.3 | 72.3 | 408.2 |
| 3_Recovery [%] | - | - | 97.0 | - | 79.0 | - | - |
| 4_Abovegr. biomass | - | 9.0* | 116.3 | 4.4 | 4.7 | 25.7 | 146.1 |
| 4_Roots | - | 35.8* | 341.6 | 4.9 | 15.5 | 40.7 | 231.4 |
| 4_Substrate | - | 9020.0* | 61.6 | 2.6 | 1.4 | 35.3 | 200.7 |
| 4_Nutrient solution | - | 1237.5^+^ | 2.1 | 3.8 | 0.1 | 0.7 | 4.0 |
| 4_Whole system | 176 | - | 521.5 | 4.5 | 21.6 | 102.5 | 582.1 |
| 4_Recovery [%] | - | - | 130.3 | - | 104.8 | - | - |
| 5_Abovegr. biomass | - | 10.7* | 227.7 | 4.8 | 10.2 | 30.9 | 173.9 |
| 5_Roots | - | 21.5* | 311.0 | 5.1 | 14.6 | 28.1 | 157.9 |
| 5_Substrate | - | 9380.0* | 59.9 | 2.1 | 1.1 | 39.4 | 221.6 |
| 5_Nutrient solution | - | 1246.0^+^ | 4.5 | 4.8 | 0.2 | 0.6 | 3.6 |
| 5_Whole system | 178 | - | 603.1 | 4.7 | 26.0 | 99.1 | 556.9 |
| 5_Recovery [%] | - | - | 120.5 | - | 100.8 | - | - |
| 6_Abovegr. biomass | - | 6.6* | 110.6 | 4.6 | 4.6 | 20.9 | 118.5 |
| 6_Roots | - | 23.5* | 315.9 | 5.0 | 14.7 | 30.8 | 174.7 |
| 6_Substrate | - | 9370.0* | 78.9 | 2.2 | 1.5 | 50.4 | 286.5 |
| 6_Nutrient solution | - | 1194.0^+^ | 3.2 | 4.5 | 0.1 | 0.6 | 3.7 |
| 6_Whole system | 176 | - | 508.7 | 4.5 | 21.0 | 102.7 | 583.3 |
| 6_Recovery [%] | - | - | 101.7 | - | 81.2 | - | - |

*Mass of fraction, ^+^Volume of fraction

^15^N content of fertilizer: 5.527 at. %, total N content of fertilizer (pots 1 and 2): 300 mg N, total N content of fertilizer (pots 3 and 4): 400 mg N, total N content of fertilizer (pots 5 and 6): 500 mg N

^15^N content of grass seed: 0.3732 at. %, total N content of grass seed: 0.0467 mg N
