# Supplementary material for: Effects of grass species and grass growth on atmospheric nitrogen deposition to a bog ecosystem surrounded by intensive agricultural land use
Source: Ecol Evol. 2015 Jun 3;5(13):2556–71. doi: 10.1002/ece3.1534 (PMC4523353; doi:10.1002/ece3.1534)
Supplement: Supplementary file 3 [file ece30005-2556-sd3.docx]

*Suppl. Tab.3. Duration of experiments, volume, N contents and N recoveries, ^15^N contents and ^15^N recoveries, and calculated N deposition for the single fractions of Eriophorum vaginatum in 2012 (exp. = experiments (pre-cultivation and exposition in the field)).*

| Pot number and | Duration of exp. | Mass/Volume | N content | ^15^N | ^15^N excess | Deposited N | N allocation rate |
| --- | --- | --- | --- | --- | --- | --- | --- |
| fraction | [days] | [g DM pot^-1^; mL pot^-1^] | [mg] | [at. %] | [mg] | [mg pot^-1^] | [µg d^-1^ pot^-1^] |
| 1_Abovegr. biomass | - | 2.4* | 5.4 | 2.7 | 0.4 | 2.9 | 12.4 |
| 1_Roots | - | 12.9* | 25.9 | 2.8 | 0.6 | 13.6 | 57.8 |
| 1_Substrate | - | 9000.0* | 96.5 | 0.5 | 0.1 | 93.8 | 399.0 |
| 1_Nutrient solution | - | 494.0^+^ | 0.2 | 0.8 | 0.0 | 0.2 | 0.9 |
| 1_Whole system | 235 | - | 128.0 | 1.1 | 0.9 | 110.5 | 470.1 |
| 1_Recovery [%] | - | - | 126.7 | - | 17.5 | - | - |
| 2_Abovegr. biomass | - | 2.0* | 12.2 | 2.6 | 0.3 | 7.0 | 29.6 |
| 2_Roots | - | 15.6* | 57.5 | 2.6 | 1.3 | 33.0 | 138.8 |
| 2_Substrate | - | 9000.0* | 100.0 | 0.5 | 0.1 | 97.9 | 411.4 |
| 2_Nutrient solution | - | 522.0^+^ | 0.2 | 0.8 | 0.0 | 0.2 | 0.7 |
| 2_Whole system | 238 | - | 169.9 | 1.3 | 1.6 | 138.2 | 580.6 |
| 2_Recovery [%] | - | - | 168.2 | - | 31.7 | - | - |
| 3_Abovegr. biomass | - | 6.9* | 19.3 | 4.6 | 0.8 | 3.5 | 14.7 |
| 3_Roots | - | 41.4* | 115.0 | 4.6 | 4.8 | 21.3 | 89.6 |
| 3_Substrate | - | 9000.0* | 77.6 | 1.1 | 0.5 | 67.3 | 282.6 |
| 3_Nutrient solution | - | 495.0^+^ | 0.7 | 4.2 | 0.0 | 0.3 | 0.8 |
| 3_Whole system | 238 | - | 212.7 | 3.3 | 6.2 | 92.2 | 387.6 |
| 3_Recovery [%] | - | - | 81.5 | - | 46.3 | - | - |
| 4_Abovegr. biomass | - | 4.6* | 37.7 | 4.7 | 1.6 | 6.4 | 26.9 |
| 4_Roots | - | 14.3* | 122.1 | 4.7 | 5.2 | 20.7 | 87.1 |
| 4_Substrate | - | 9000.0* | 69.9 | 1.1 | 0.5 | 59.4 | 249.6 |
| 4_Nutrient solution | - | 540.0^+^ | 0.5 | 4.0 | 0.0 | 0.2 | 0.7 |
| 4_Whole system | 238 | - | 230.2 | 3.6 | 7.4 | 86.7 | 364.2 |
| 4_Recovery [%] | - | - | 88.2 | - | 55.2 | - | - |
| 5_Abovegr. biomass | - | 9.0* | 78.2 | 4.9 | 3.6 | 9.2 | 38.5 |
| 5_Roots | - | 25.1* | 209.1 | 4.9 | 9.5 | 25.3 | 105.7 |
| 5_Substrate | - | 9000.0* | 67.4 | 1.9 | 1.0 | 47.3 | 197.7 |
| 5_Nutrient solution | - | 508.0^+^ | 0.8 | 4.0 | 0.0 | 0.2 | 1.0 |
| 5_Whole system | 239 | - | 355.6 | 4.3 | 14.1 | 81.9 | 342.9 |
| 5_Recovery [%] | - | - | 80.6 | - | 62.2 | - | - |
| 6_Abovegr. biomass | - | 12.2* | 49.5 | 4.9 | 2.3 | 5.6 | 23.4 |
| 6_Roots | - | 77.4* | 314.9 | 4.9 | 14.4 | 36.2 | 151.4 |
| 6_Substrate | - | 9000.0* | 67.9 | 1.7 | 0.9 | 50.0 | 209.1 |
| 6_Nutrient solution | - | 519.0^+^ | 0.6 | 3.9 | 0.0 | 0.2 | 0.8 |
| 6_Whole system | 239 | - | 433.0 | 4.4 | 17.6 | 91.9 | 384.7 |
| 6_Recovery [%] | - | - | 98.2 | - | 77.5 | - | - |

*Mass of fraction, ^+^Volume of fraction

^15^N content of fertilizer: 5.527 at. %, total N content of fertilizer (pots 1 and 2): 100 mg N, total N content of fertilizer (pots 3 and 4): 260 mg N, total N content of fertilizer (pots 5 and 6): 440 mg N

^15^N content of grass seed: 0.3701 at. %, total N content of grass seed: 0.0635 mg N
